# Comparing Clinical Outcomes of Microdiscectomy, Interspinous Device Implantation, and Full-Endoscopic Discectomy for Simple Lumbar Disc Herniation

**DOI:** 10.3390/jcm14061925

**Published:** 2025-03-13

**Authors:** Chien-Ching Lee, Ruey-Mo Lin, Wei-Sheng Juan, Hao-Yu Chuang, Hung-Lin Lin, Cheng-Hsin Cheng, Chun-Hsu Yao

**Affiliations:** 1 Department of Anesthesia, An Nan Hospital, China Medical University, Tainan 70965, Taiwan; 2 Department of Medical Sciences Industry, Chang Jung Christian University, Tainan 71101, Taiwan; 3 Department of Orthopedics, An Nan Hospital, China Medical University, Tainan 70101, Taiwan; 4 Department of Neurosurgery, An Nan Hospital, China Medical University, Tainan 70965, Taiwan; 5 Department of Neurosurgery, China Medical University Beigang Hospital, Yunlin 65152, Taiwan; 6 Department of Neurosurgery, China Medical University Hospital, Taichung 40447, Taiwan; 7 Graduate Institute of Medical Sciences, Chang Jung Christian University, Tainan 71101, Taiwan; 8 Department of Biomedical Imaging and Radiological Science, China Medical University, Tainan 70965, Taiwan; 9 School of Chinese Medicine, China Medical University, Tainan 70965, Taiwan; 10 Biomaterials Translational Research Center, China Medical University Hospital, Taichung 40447, Taiwan; 11 Department of Biomedical Informatics, Asia University, Taichung 41354, Taiwan

**Keywords:** degenerative disc disease, discectomy endoscopic, interspinous process devices, lumbar disc herniation, microdiscectomy, percutaneous endoscopic lumbar discectomy

## Abstract

**Background/Objectives**: The treatment for lumbar disc herniation (LDH) is surgical discectomy. This surgery may enhance spinal instability and exacerbate disc degeneration. The most common treatment options include microdiscectomy (MD), interspinous process device (IPD) implantation, and percutaneous endoscopic lumbar discectomy (PELD). As few studies have compared these three procedures, this study focused on collecting data on the clinical, functional, and imaging outcomes of surgery for symptomatic LDH. **Methods**: This is a retrospective, transverse, and analytical study, with a total of 383 patients who received operations for symptomatic LDH between 2018 and 2022. Medical information from the charts of these patients was collected. The results were followed up on for a minimum of one year by collecting responses from several questionnaires and clinical data, including patients’ scores on the visual analogue scale (VAS), Oswestry Disability Index (ODI), and symptomatic improvement score (SIS), as well as wound size, blood loss, hospital stay, postoperative disc change, and complications. **Results**: At the end of data collection, the VAS and ODI scores all showed significant improvement following these three procedures (*p* < 0.01). The SISs were all ranked as good (8.1, 8.5, and 7.9) post-surgery. PELD was a minimally invasive procedure that resulted in the smallest wound size (0.82 cm), minimal blood loss (21 mL), and a short hospital stay (4.2 days). A substantial pre-/postoperative change in disc height was noted in the MD (−17%) and PELD (−15%) groups. The complication rates were similar among the three groups (3%, 5%, and 5.6%). **Conclusions**: IPD implantation and PELD yielded outcomes comparable to those of conventional MD for symptomatic relief and functional recovery. Although the complication rates were similar, the postoperative complications were quite different from those of the other procedures. PELD resulted in rapid recovery and minimal invasion, and IPD implantation showed a good ability to preserve disc height and spinal stability; however, the clinical relevance of these findings in disc degeneration remains controversial.

## 1. Introduction

The increasing ageing population has encountered substantial challenges in spinal medical care. Lumbar disc herniation (LDH) is a critical spinal disease primarily treated with surgical decompression [1]. The symptoms of LDH may present as radicular signs, neurogenic claudication, and low back pain, and they frequently occur in individuals over 50 years of age [2]. The options for the management of LDH include conservative treatment (such as epidural steroid/morphine injections), surgical decompression with microdiscectomy (MD), the implantation of an interspinous process device (IPD), and percutaneous endoscopic lumbar discectomy (PELD) [3]. **Surgical decompression is indicated if conservative treatments fail** [4,5,6]. Definitive decisions regarding surgical methods for simple LDH remain controversial [7,8,9]. Since Caspar and Yasargil developed the method of surgical discectomy with microscopic assistance in 1977, MD has become the gold standard technique for LDH [10,11,12,13]. Recently, some studies have stated that the disruption of the paravertebral muscles in the subperiosteal approach and the destruction of the lamina may induce spinal instability and failed-back syndrome [14,15]. A randomized controlled study showed no significant differences in functional outcomes and recovery times between open and minimally invasive MD [16,17]. IPDs have been used for decades and may be designed as a static or dynamic component according to the material, manufacturing, and design [7,18,19,20,21,22]. In 1986, a hard dynamic stabilized system was created to decrease postoperative spinal instability with an interspinous mass and reduce the angle of extension; tension bands were used to fix the implanted graft in the adjacent spinous processes [23]. The considerable indications of application are extensive and can treat spinal canal stenosis, symptomatic facet syndrome for low back pain, discogenic pain, radiculopathy by disc herniation, and an unstable spine [18,19,24,25]. This technique is reversible in cases of recurrent or persistent low back pain. The stabilization system was removed and replaced with a rigid fixation system. In 1997, Minns first presented an interspinous silicone implant for dynamic lumbar stabilization [26]. Since then, an increasing number of soft dynamic system products have been created for lumbar degenerative disc surgery [9,27]; however, their associated use with conventional MD is controversial, requiring a more evidenced database [22,25]. This, combined with its non-specific and ‘useful-for-all’ characteristics, has made its use debatable among spinal operators [8,25]. PELD with a 1 cm incision has been widely performed for LDH and the stenosis of the spinal canal [28]. Its advantages include minimal wounds, less destruction, less blood loss, and a short operative time [29,30]. However, in endoscopic percutaneous spine surgery with one small linear incision, its unidirectional axis and working channel limit the operative view and decompressive area [31]. Several reports have found similar clinical outcomes in terms of the safety and effectiveness of PELD and MD procedures in the management of LDH [32,33]; however, analyses and reviews focusing on the comparison of MD, IPD implantation, and PELD have not yet been widely reported. Therefore, this study tried to estimate the clinical and functional outcomes of these three different methods for the management of LDH.

## 2. Materials and Methods

In total, 383 patients (average age, 59.9 years; range, 18–82 years) who received spine surgery for symptomatic LDH between January 2018 and December 2022 were selected for evaluation. The inclusion criteria were symptoms of persistent and severe radiculopathy caused by LDH, **failure of conservative treatment** for 3–6 months, confirmation of diagnosis with spine magnetic resonance imaging (MRI), treatment of a patient by a conventional discectomy of the MD/IPD/PELD procedures, and a follow-up of 1 year. The exclusion criteria included the revelation of an objective neurological deficit via clinical evaluation; revelation of other associated pathological problems such as tumours, infection, spine fracture, spondylosis, spinal stenosis, and facet syndrome via imaging studies (MRI or X-ray); and a history of previous spine surgery.

### Clinical Data Collection

The data analysis of the variables in this study was divided into four steps. First, basic data were collected from the patients’ medical records, such as age, sex, cigarette use, body mass index (BMI), surgical spinal level, and type of operation. Second, data were obtained from the medical charts of patients who received operations, including wound size, surgical time, amount of bleeding, admission days, and postoperative complications with or without further re-intervention, if any, together with the time period from the first surgery to the next intervention.

**Figure 1:**
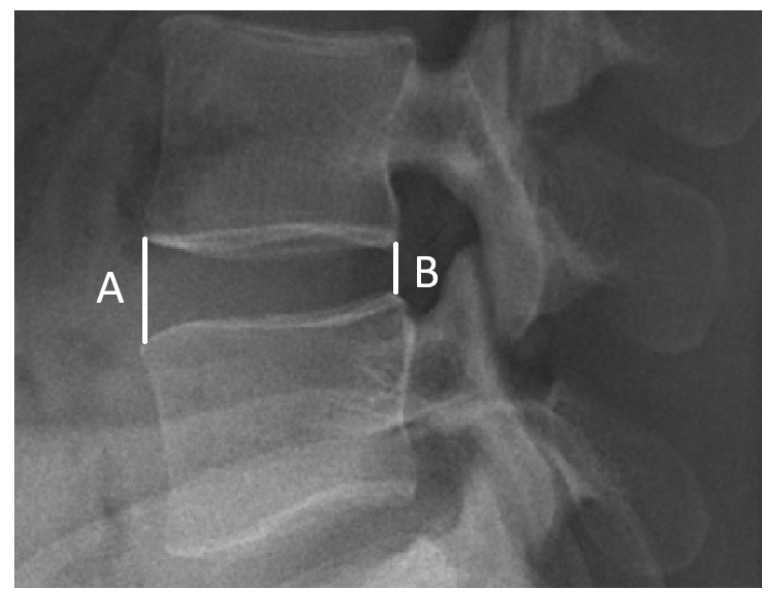
Measurement of disc height. Calculation of disc height via Dabbs’ method (A + B/2).

Thirdly, a radiographic assessment was conducted. For all cases studied, a lateral upright lumbar radiograph was obtained before and 6 months after the operations. To evaluate disc differences, the intervertebral disc height (IDH) was calculated preoperatively/postoperatively using Dabbs’ method [34,35]. The IDH was recorded as the average sum at the height of the anterior (A) and posterior (B) edge of the disc. The calculated formula was ((A + B)/2) (Figure 1). Average IDH values were compared among the three groups. Furthermore, the correlation between the clinical outcomes and changes in postoperative IDH was evaluated. All of the imaging calculations were performed by the same radiologist.

Finally, the final clinical response after surgical intervention was evaluated using consistent surveys frequently used in spinal surgical evaluation. The questionnaires included the Oswestry Disability Index (ODI) to estimate the degree of disability and the visual analogue scale (VAS) to record the pain strength. The VAS score was assessed from 0 (no pain) to 10 (worst pain) using a millimetre rule. The data of the ODI are presented as a percentage from 0 to 100% and recorded with standardized measurements. Questionnaires were collected for both the preoperative status and postoperative follow-up. A symptomatic improvement score (SIS) was also designed to better understand the symptomatic improvement before and after surgery to a greater degree. Satisfaction with the surgery ranged from 0 to 10 on the SIS by the patients’ self-judgement or a caretaker’s inference (0–2: poor; 3–5: satisfactory; 6–8: good; and 9–10: excellent).

## 3. Results

The average age of the patients was 59.9 (59.8% male and 40.2% female). Of the patients, 200 underwent conventional MD, 76 underwent IPD implantation, and 107 underwent PELD. The baseline data and clinical profiles of these three groups revealed no statistically significant differences according to their *p*-values (>0.05). The data of the VAS, ODI, and SIS were also recorded. The data showed that the VAS and ODI scores improved meaningfully in all three groups after operation (*p* < 0.01) (Table 1). The satisfaction of patients was scored using the SIS and was 8.1, 8.5, and 7.9 out of 10 for MD, IPD, and PELD, respectively (*p* > 0.05). The operative spinal levels are listed in Table 1.

According to our medical records, the mean operative times were 126.35 ± 38.5, 171.59 ± 56.98, and 127.92 ± 47.64 min in the MD, IPD, and PELD groups, respectively, as shown in Table 2. The average operative time in the IPD patients was significantly longer than in the MD and PELD patients (*p* < 0.001). Nevertheless, the operative blood loss (21 ± 23.13 mL) and length of hospital stays (4.2 ± 2.35 days) in the PELD patients were significantly lower than those in the MD patients (73.1 ± 102.25 mL and 5.5 ± 3.59 days, respectively) and IPD group (164.74 ± 180.75 mL and 6.97 ± 4.22 days, respectively). The operative wound sizes were similar in the MD and IPD groups. The PELD group demonstrated the smallest wound size (average of 0.82 mm). The IDH was measured separately for each surgical lumbar level, both preoperatively and postoperatively. The IDH decreased postoperatively in the MD (−17%) and PELD (−15%) groups. In contrast, the mean IDH in the IPD group increased by 3%.

The total complication rate was 4% (*n* = 8), 5.2% (*n* = 4), and 7.5% (*n* = 8) for the MD, IPD, and PELD groups, respectively (Table 2). No difference was noted among the three groups. Surgical infection (SI) is a common complication in spinal surgery. Four patients each from the MD and IPD groups underwent debridement and antibiotic treatment for postoperative infections. No infections were observed in the PELD group. Residues/recurrences of lumbar herniated discs were present in three patients of the MD group and four patients of the PELD group. No secondary operations were performed for recurrent disc rupture in the IPD group.

Neural damage (drop foot) may be encountered in spine surgery. In this study, it occurred in only one patient in the MD group due to nerve traction. The patient recovered completely after six months of rehabilitation and medication. The PELD group presented four cases of dural tears, whereas no incidental durotomy happened in the other groups. Four patients with dural openings in the PELD group underwent open surgery for dural repair. In our review, six patients in the MD group, one in the IPD group, and three in the PELD group underwent fusion surgery due to spinal instability within 1 year of the follow-up. The specific data for secondary surgery are listed in Table 3.

## 4. Discussion

In most circumstances, patients were in favour of a minimally invasive approach to surgical intervention because of its fast postoperative recovery times [36,37]. Whether minimally invasive spinal surgery (MISS) can offer better clinical results compared to traditional spine operations has long been a concern for spine operators [38]. Therefore, there is a growing interest in less invasive therapeutic solutions as alternatives to lumbar fusion. Other types of implants with lower local aggressiveness, such as IPDs, are also included in this context [39]. PELD is a recently developed MISS technique. Reports from case series have revealed satisfactory clinical outcomes in the management of LDH [15,40,41,42]. Thus far, few reports have compared the operative outcomes of patients undergoing PELD, IPD implantation, and open MD. Hence, this study attempted to understand the clinical outcomes of these three popular spine procedures for symptomatic LDH.

The collected results showed that the PELD, IPD, and MD groups all achieved great progress in their postoperative VAS and ODI scores. In this retrospective study, the data revealed that MD, PELD, and IPD implantation can reach satisfactory and similar clinical results at a 1-year follow-up. In particular, PELD showed faster postoperative recovery, which was reflected in fewer hospital days and less operative bleeding with limited wound size (*p* < 0.01). All three groups achieved high levels of satisfaction. While PELD and IPD implantation may have higher initial costs, their suitability depends on the patient’s condition. Microdiscectomy is generally the most cost-effective option for disc herniation. Both immediate surgical costs and long-term recovery expenses should be considered when choosing the best treatment [43]. The conventional MD procedure remained an efficient and well-recovered procedure in our series.

The PELD procedure presents a similar interlaminar approach to MD [26,35]. This distinguishing feature requires endoscopic procedures to take advantage of MD [23,35]. Nevertheless, this MISS technique necessitates a great learning curve for young beginners [36]; skilled and well-trained surgeons may produce different clinical outcomes. Several reports have shown that the operative time for endoscopic surgery is longer than that for MD [25,37]. In our data, the average operative time was similar for the MD and PELD groups (126.35 min and 127.92 min), demonstrating that PELD has no benefits with regard to shortening the operative time; however, IPD implantation resulted in the longest operative time, more blood loss, and larger wound size, which may be caused by the larger operative area approach and instrument implantation. In the consideration of surgical procedures, the creation of a working area, operative hemostasis, and implant adjustment are time-intensive. The period of hospital admission in the PELD group was significantly shorter than that for the other two groups, **possibly** indicating that patients who underwent spinal endoscopic surgery were able to undergo rapid exercise, rehabilitation, walking, and an earlier return to daily work. These issues are very critical, particularly for young patients who are willing to return to their work earlier and older people who have a better quality of life. Our study showed that patients receiving an IPD paid higher admission fees than those receiving MD and PELD techniques. This may be related to the health insurance system of Taiwan, where this study was conducted.

There was no difference in the total complication rate among all of the groups in our study (*p* > 0.05). Four instances of SI occurred in the MD and IPD groups, whereas no such complications were found in the PELD group. These infectious complications were treated with surgical debridement, cleaning, and antibiotic injections, with good recovery. The data demonstrate the benefit of a minimally invasive procedure in decreasing wound-related problems. The rate of a recurrent disc is a substantial concern for endoscopic spine procedures, with a possibility ranging from 0 to 6.9% [44,45,46]. Patients that received the PELD procedure are considered as being at a higher risk of re-ruptured disc herniation owing to the restricted flexibility in management and the limited operative field. Although the recurrent rates in the conventional MD and PELD groups were similar at the 1-year follow-up, studies with longer periods are required to validate this observation. Therefore, it is noteworthy that there were no secondary operations for recurrent ruptured discs in the IPD group. IPD implantation helps to reduce spinal extension motion and disc/facet joint stresses [47]. This may be one of the reasons for its lower recurrence rates; however, it has special complications, such as implant dislocation, malposition, or fracture of the spinal process [48].

In the MD group, there was one case of foot drop immediately after the operation due to nerve traction. The patient received steroids and underwent rehabilitation without surgical intervention. The neurological deficiency recovered well after six months of treatment. There were no postoperative neurological deficits in the PELD or IPD group of the present study.

A total of 27% of patients who undergo discectomy present postdiscectomy syndrome or failed-back syndrome, requiring a second surgery within 10 years of the first intervention [48,49]. One possible solution with which to avoid postdiscectomy syndrome is to perform lumbar fusion instrumented with pedicle screws during surgical discectomy. Unsatisfactory long-term pain relief after lumbar MD occurred in 8–25% of examined patients [50,51]. Segmental instability is a critical issue that contributes to failed-back syndrome. Extensive operative techniques and bony destruction during MD may increase the possibility of secondary segmental instability [52]. In our series, six cases (3%) in the MD group and three (2.8%) in the PELD group underwent spinal fusion operation within a follow-up of 1 year due to spinal instability. Only one patient (1.3%) in the IPD group underwent a secondary fusion operation. The purpose of an IPD is to keep segmental stability, preserve the focal segment mobility, and minimize the degenerative effects of adjacent segments. This may have resulted in lower postoperative segmental instability [53].

This study had a few limitations, including a relatively small number of samples and a short follow-up period. Hence, more randomized controlled and prospective studies with designs for the PELD and IPD procedures are necessary to confirm our findings. Additionally, only patients with LDH were discussed, whereas those with spinal stenosis or mild-to-moderate spinal instability were excluded. Therefore, there are no reasons to suppose that the conclusions of this research are reasonable for current surgical decisions for LDH patients.

## 5. Conclusions

The PEID and IPD procedures yielded comparable outcomes to traditional MD for pain improvement and clinical outcomes 1 year postoperation. In addition, PELD enables shorter hospital stays and faster recovery in the immediate postoperative period. Although an IPD leads to a prolonged operative time, more bleeding, and extended hospital stays, its long-term patient satisfaction is similar to that of other procedures. The subsequent effects of a limited range of motion and decreased disc pressure are a concern. The MD technique is an effective and efficient operation for symptomatic LDH. As this was a retrospective study with only 1 year of follow-up, more randomized controlled and prospective trials would be required to validate our observations.

## 6. Limitation

This study’s small sample size limits the generalizability of the results. A larger cohort would provide more reliable data on the efficacy and safety of the treatments. Additionally, the one-year follow-up may not be long enough to capture long-term outcomes or complications. A longer follow-up would better assess treatment durability and late complications.

Future research with more cases and extended follow-up is needed to fully evaluate these procedures’ long-term effectiveness and safety.

## Figures and Tables

**Table 1:** Baseline clinical characteristics and demographic data.

	MD	IPD	PELD
Age (y/o)	58.1 ± 17.43	53.5 ± 14.10	53.5 ± 15.55
Male/female	121/79	42/34	66/41
Smoking (%)	35%	25%	21%
BMI	31.8 ± 4.36 *	28.4 ± 5.67 *	27.5 ± 4.22 *
Preop./postop. VAS *	8.22/1.39 *	7.35/1.89 *	8.35/1.34 *
Preop./postop. ODI (%)	75.4/18.7 *	73.5/16.3	71.2/12.4 *
SIS	8.1	8.5	7.9
Op. Level (N)			
L1/2	3	2	
L2/3	12	7	5
L3/4	22	10	6
L4/5	91	57	59
L5/S1	72	0	37

BMI: body mass index; IPD: interspinous process device; MD: microdiscectomy; N: number; ODI: Oswestry Disability Index; PELD: percutaneous endoscopic lumbar discectomy; SIS: symptom improvement score; VAS: visual analogue scale; and * *p*-value < 0.001.

**Table 2:** Operative record information.

	MD	IPD	PELD
Operative time (mins)	126.35 ± 38.5	171.59 ± 56.98	127.92 ± 47.64
Blood loss (mL) *	73.1 ± 102.25	164.74 ± 180.75	21 ± 23.13
Wound size (cm)	5.89 ± 3.76	6.74 ± 2.86	0.82 ± 0.76
Hospital stays (days)	5.5 ± 3.59	6.97 ± 4.22	4.2 ± 2.35
Medical cost (NT$) *	32,845 ± 3458	150,984 ± 8354	107,304 ± 5748
Preop./postop. disc height (mm)	10.2/8.43 *	9.12/9.35	8.23/7.03 *
Change in IDH (%)	−17%	+3%	−15%
Complication rate (%)	4%	5.2%	7.5%

IDH: intervertebral disc height; IPD: interspinous process device; MD: microdiscectomy; PELD: percutaneous endoscopic lumbar discectomy; and * *p*-value < 0.001.

**Table 3:** Cause of secondary operation for three studied groups.

	MD	IPD	PELD
SI	4	4	-
Recurrent rupture disc	3	-	4
Drop foot	1	-	-
Dura tear	-	-	4
Spine instability	6	1	3

IPD: interspinous process device; MD: microdiscectomy; PELD: percutaneous endoscopic lumbar discectomy; and SI: surgical infection.

## Data Availability

The original contributions presented in the study are included in the article, further inquiries can be directed to the corresponding authors.
